# Differential Susceptibility of Fetal Retinal Pigment Epithelial Cells, hiPSC- Retinal Stem Cells, and Retinal Organoids to Zika Virus Infection

**DOI:** 10.3390/v15010142

**Published:** 2023-01-01

**Authors:** Deisy Contreras, Gustavo Garcia, Melissa Kaye Jones, Laura E. Martinez, Akshaya Jayakarunakaran, Vineela Gangalapudi, Jie Tang, Ying Wu, Jiagang J. Zhao, Zhaohui Chen, Arunachalam Ramaiah, Irena Tsui, Ashok Kumar, Karin Nielsen-Saines, Shaomei Wang, Vaithilingaraja Arumugaswami

**Affiliations:** 1 Board of Governors Regenerative Medicine Institute, Cedars-Sinai Medical Center, Los Angeles, CA 90048, USA; 2 Department of Biomedical Sciences, Cedars-Sinai Medical Center, Los Angeles, CA 90048, USA; 3 Department of Molecular and Medical Pharmacology, University of California, Los Angeles, CA 90095, USA; 4 Genomics Core, Cedars-Sinai Medical Center, Los Angeles, CA 90048, USA; 5 Alpine BioTherapeutics Corporation, 11107 Roselle Street, Suite 210, San Diego, CA 92121, USA; 6 Tata Institute for Genetics and Society, Center at inStem, Bangalore 560065, India; 7 Retina Division, Department of Ophthalmology, University of California at Los Angeles, Los Angeles, CA 90095, USA; 8 Department of Ophthalmology, Visual and Anatomical Sciences, Wayne State University, Detroit, MI 48201, USA; 9 Department of Pediatrics, University of California, Los Angeles, CA 90095, USA; 10 Eli and Edythe Broad Center of Regenerative Medicine and Stem Cell Research, University of California, Los Angeles, CA 90095, USA

**Keywords:** Zika virus, human fetal retinal pigment epithelial cells, human iPSC-derived retinal stem cells, retinal organoids, congenital eye disease, apoptosis, nucleoside analogue

## Abstract

Zika virus (ZIKV) causes microcephaly and congenital eye disease. The cellular and molecular basis of congenital ZIKV infection are not well understood. Here, we utilized a biologically relevant cell-based system of human fetal retinal pigment epithelial cells (FRPEs), hiPSC-derived retinal stem cells (iRSCs), and retinal organoids to investigate ZIKV-mediated ocular cell injury processes. Our data show that FRPEs were highly susceptible to ZIKV infection exhibiting increased apoptosis, whereas iRSCs showed reduced susceptibility. Detailed transcriptomics and proteomics analyses of infected FRPEs were performed. Nucleoside analogue drug treatment inhibited ZIKV replication. Retinal organoids were susceptible to ZIKV infection. The Asian genotype ZIKV exhibited higher infectivity, induced profound inflammatory response, and dysregulated transcription factors involved in retinal organoid differentiation. Collectively, our study shows that ZIKV affects ocular cells at different developmental stages resulting in cellular injury and death, further providing molecular insight into the pathogenesis of congenital eye disease.

## 1. Introduction

Zika virus (ZIKV) is a teratogenic vector-borne pathogen that causes detrimental congenital eye abnormalities along with other congenital birth defects, such as microcephaly, intrauterine growth restriction, and sensorineural hearing loss [1,2]. ZIKV is a member of the *Flaviviridae* family, comprised of other relevant human pathogens such as West Nile, Japanese Encephalitis, Dengue, and St. Louis Encephalitis viruses. ZIKV was first isolated from humans in 1952 in Uganda and Tanzania, and its main mode of transmission is vectorial through the bite of an infected *Aedes* species mosquito (*Aedes aegypti* or *Aedes albopictus* [3]. However, studies provide increasing evidence that ZIKV can be spread sexually from men or women, and through transplacental transmission in infected mothers [4]. ZIKV may gain access to the fetal eye through hematogenous route and direct ocular exposure to infected amniotic fluid in utero resulting in congenital eye diseases [5,6]. Approximately 80% of adults infected with ZIKV remain asymptomatic, while the remaining infected population exhibit mild symptoms, such as short-lived fever, rash, and joint pain, all of which are rarely life threatening [3]. ZIKV has also been linked to Guillain-Barré syndrome, which can damage nerve cells and cause temporary paralysis in infected adults [7,8,9].

Congenital eye abnormalities can occur at a variable rate worldwide and approximately 20 million children under the age of 18 years suffer from some type of congenital eye disorder, resulting in more than 1.4 million cases of blindness [10,11,12]. Human infections known to cause congenital birth defects include *Toxoplasma gondii*, Rubella virus, Cytomegalovirus (CMV), and Herpes Simplex Virus 1 (HSV-1), referred to as TORCH infections, and can be acquired by an intrauterine route [4]. A repertoire of clinical studies on ZIKV infected fetuses have found that infection can lead to characteristic eye anomalies, such as macular pigment mottling, chorioretinal atrophy, optic nerve hypoplasia, optic nerve pallor, and blindness during the first or second trimester of pregnancy [5,13,14,15,16]. Approximately 35% of microcephalic babies showed posterior pole pigmentary clumping and chorioretinal atrophy in the eyes [5]. ZIKV also less commonly causes other structural eye anomalies such as microphthalmia and coloboma, cataracts, glaucoma, and intraocular calcifications in the eye [17,18]. However, the molecular mechanisms underlying the structural and developmental eye anomalies caused by ZIKV infection are not well understood.

Immunocompromised mouse model systems have been utilized to reproduce key features of human ZIKV infection in mice, including neuronal and ocular tissue tropism [19,20,21,22,23,24]. ZIKV has a broader cell tropism than other flaviviruses [25,26]. A study using an *Ifnar1*^−/−^ knockout mouse model system demonstrated the presence of ZIKV in different parts of the eye, including the cornea, retinal epithelial cell layer, and tear fluid, which further contributed to conjunctivitis and uveitis [6]. However, congenital eye disorder was not observed in *Ifnar1*^+/−^ fetuses of C57BL/6 *Ifnar1*^−/−^ infected mothers [22]. In addition, retinal damage caused by Zika virus has also been investigated in a Zebrafish model system [27].

A previous study reported that in vitro ZIKV infection of human retinal pigment epithelial (RPE) cells caused detrimental effects on membrane permeability by disrupting cell-to-cell junctions [28]. Meta-analysis of transcriptome profiles generated from ZIKV-infected human primary RPE cells revealed the perturbation of SH3/SH2 adaptor activity, lipid and ceramide metabolism, and embryonic organ development [29]. We have observed that Zika virus infection can activate Hippo signaling pathway in RPE cells, which can play an important role in regulating viral replication and inflammatory responses [24]. Another report showed that ZIKV causes ocular complications, such as chorioretinal atrophy, by infecting cells lining the blood–brain barrier and the retinal pigment epithelium [29]. Collectively, these findings indicate that retinal cells are the primary target of ZIKV in the eye. To our knowledge, how ZIKV infection affects the developing human retina has not been studied. Fetal ocular infectious cell culture model systems will be more relevant to study ZIKV pathogenesis during the early developmental stages of the eye.

In this study, we utilized three models systems (1) second-trimester human fetal retinal pigment epithelial (FRPE) progenitor cells, (2) human induced pluripotent stem cell (iPSC)-derived retinal stem cells (iRSCs), and (3) human iPSC-derived retinal organoids to investigate the pathogenic processes of congenital ZIKV infection in vitro by examining the initial cellular response to infection and injury. We found that ZIKV infection alters cell viability by inducing programmed cell death in FRPE cells. Global transcriptomics and proteomics analyses of infected FRPE cells revealed dysregulation of cell survival pathways and VEGF signaling, and activation of innate immune and inflammatory response genes. Antiviral drug screening against ZIKV revealed that a nucleoside analogue, 6-Azauridine, inhibited viral replication and blocked ZIKV-mediated cell death in FRPE cells. iRSCs show reduced susceptibility to ZIKV infection possibly due to low-level expression of several ZIKV entry receptors and co-factors. Neural retina and RPE progenitor cells of developing retinal tissue (retinal organoids) were susceptible to ZIKV infection. The virulent Asian genotype ZIKV strain (PRVABC59) replicated more efficiently in mature retinal organoids as compared to the low pathogenic African genotype strain (MR-766).

## 2. Materials and Methods

### 2.1. Cells

Human fetal retinal pigment epithelial (FRPE) cells (kindly provided by Dr. Guoping Fan at UCLA), Vero cells (ATCC), and ARPE-19 cells (ATCC) were grown in DMEM high glucose medium supplemented with 10% fetal bovine serum and 1% penicillin/streptomycin. FRPEs were originally isolated from fetal retina (~20-week-old). The cells were incubated at 37 °C supplemented with 5% CO_2_ and passaged when 90% confluence was reached at approximately every third day using 0.05% Trypsin with 0.53 mM EDTA (Corning, Glendale, CA, USA).

Induced pluripotent stem cells (iPSCs)-derived retinal stem cells (iRSCs): The iPSC lines were established from BJ (ATCC^®^ CRL-2522) human fibroblast cells (ATCC, Manassas, VA, USA) and normal human dermal fibroblasts (NHDF) (UCLA iPS Core, Los Angeles, CA, USA). The iPSCs were induced into retinal stem cells (ABC010) by chemically defined conditions, as previously described [30]. In brief, induced pluripotent stem cell-derived retinal stem cells (iRSCs) were grown in DMEM/F12 media supplemented with 1% N2, 2% B27 without vitamin A, 7.5% BSA Fraction V, 1% GlutaMAX, 1% MEM non-essential amino acids, 1% of a proprietary cocktail (Alpine Biotech LLC., San Diego, CA, USA) for retinal stem cells, and 300 µM Ascorbic acid on matrigel coated flasks. Dissociation of the cells was achieved by Accutase (Life Technologies, Carlsbad, CA, USA). The expression of retinal stem cell markers was verified.

### 2.2. Differentiation of hiPSCs and Development of Retinal Organoids

We used the human iPSC line (NHDF line from UCLA iPS core) for differentiation to retinal organoids using chemically defined differentiation medium based on a published protocol [31] with modifications. In brief, on Day 0 of differentiation, hiPSCs were cultured with mTeSR1 (Stem Cell Tech., Cambridge, MA, USA) medium and 10 μM Blebbistatin (Stem Cell Tech., Cambridge, MA, USA) to induce aggregate formation. Cell aggregates were then transferred into neuro-ectoderm induction medium (NIM) containing 1:1 DMEM: F12, 1% N2 supplement (Thermo-Scientific, Waltham, MA, USA), 1× minimum essential media containing non-essential amino acids (NEAAs) and 2 µg/mL heparin (Stem Cell Tech., Cambridge, MA, USA). Medium was replaced with a 3:1 ratio of mTeSR1/neural induction medium (NIM) on Day 1 (D1), 1:1 on D2, and 100% Eye-Field Induction medium (EFIM) on D3. After 7 days of differentiation, cells are expected to acquire a retinal stem cell phenotype. On D8, cell aggregates were gently scrapped off of the plate and re-plated onto growth-factor-reduced matrigel-coated dishes (Corning, Glendale, CA, USA) containing neural induction medium. On D11–14, aggregates were suspended in a phenol-red-free and LDEV-free matrigel bubble (Corning, Glendale, CA, USA). Induction medium was switched to Retinal Differentiation Medium (RDM), comprising DMEM: F12 (3:1) supplemented with 2% B27 without vitamin A (Thermo-Scientific, Waltham, MA, USA), 1× NEAA, and 1% antibiotic-antimycotic on D16. An optional passage of organoids were done at day 21. The medium was changed daily throughout the differentiation experiment.

### 2.3. Zika Virus

The PRVABC59 (GenBank accession number KU501215) Zika virus strain of Asian genotype was used for the infection of human retinal pigment epithelial (FRPE) cells, iRSCs and 3D-retinal organoids. PRVABC59 was acquired from the Centers for Disease Control and Prevention (CDC), Atlanta, GA, USA. Early passage African genotype MR-766 ZIKV stock was obtained from ATCC, Manassas, VA, USA. Working viral stock for the specified experiments was generated by subjecting the original ZIKV strain (Passage = 3) to two additional passages in Vero cells. An established viral plaque assay was utilized to measure viral titer, as previously described [24,32].

### 2.4. Zika Viral Infection

For ZIKV infection, FRPE and iRSCs were seeded in a 24-well plate with a cell density of ~2.5 × 10^4^ cells/well. After 24-h, ZIKV inoculum, with a multiplicity of infection (MOI) of 0.1, was formulated using the base media specified for each cell type. A total of 200 µL of viral inoculum was added to each well and the plates were incubated at 37 °C with 5% CO_2_ for 2 to 4 h. After the incubation period, the base media was replaced for each cell type with their respective complete media at a volume of 1 mL per well. As for the uninfected (mock) group, each cell type received the specified cell-growth media that was concurrently used to prepare the viral inoculum, as described above. To each cell type, a mock-infected control was used for each specified time point of infection. At the end of each time point, cell lysates and RNA were harvested for downstream analyses. 

### 2.5. ZIKV Infection of Retinal Organoids

For infecting day 16 and day 29 3D-retinal organoids, organoids from each well of a 12-well plate were harvested in a sterile 2-mL tube. Each tube containing ~ 40 organoids received 1 × 10^6^ PFU of PRVABC59 or MR-766 in 100 µL volume. Tubes were inverted every 15 min during an hour incubation at 37 °C. Uninfected organoids received only 100 µL of 1× PBS. After an hour of incubation, and brief spin, organoids were transferred into matrigel bubbles. Subsequently, they were plated in the wells of 12-well plate (2 mL medium/well) and incubated at 37 °C. At 2 and 5 days post-infection (dpi), organoids were harvested for RNA or fixed with 4% paraformaldehyde for immunohistochemistry.

### 2.6. Caspase 3/7 Assay to Measure Apoptosis

Caspase-Glo 3/7 Assay (Promega, Madison, WI, USA) was performed as per the manufacturer’s protocol. At the indicated time points, mock and ZIKV infected FRPE and iRSCs were incubated with the pro-luminescent caspase-3/7 substrate for 1 h at room temperature. Subsequently, 100 µL of lysate was transferred to a white 96-well microtiter plate for reading the luminescence signal using a luminometer (Glomax Microplate Luminometer, Promega, Madison, WI, USA).

### 2.7. Cell Viability Assay

CellTiter-Glo Luminescent Cell Viability Assay (Promega, Madison, WI, USA) was performed per the manufacturer’s protocol. In brief, the CellTiter-Glo reagent was directly added to both infected and uninfected cells at the designated time points. The addition of this reagent resulted in the lysis of the cells with the release of a luciferase signal, which was measured by a luminometer. The luminescence signal is a direct measurement of ATP present in the viable cells in culture.

### 2.8. Proteomic Sample Preparations and Liquid Chromatography Mass Spectrometry (LC-MS/MS) Analysis

For proteomics, biological quadruplicates were prepared and pooled in duplicates for mass spectrometry. After FRPE cells were lysed in 80 mM Tris-HCl, 4% SDS, 100 mM DTT at pH 7.4, 30 μg of protein was quantified for each sample and further digested using the FASP Protein Digestion Kit (Expedeon, San Diego, CA, USA), as per manufacturer’s instructions.

Liquid chromatography tandem mass spectrometry (LC-MS/MS) system was set up on a Transcend II LX-2 HTLC system connected to an Q exactiveplus equipped with an HESI ion source (QE, Thermo Scientific, Waltam, MA, USA). A LC-MS/MS setup was used to separate the tryptic digested peptides with mobile phases A (0.1% formic acid) and B (0.1% formic acid in 100% acetonitrile). The tryptic peptide mixture (20 μg) was loaded onto a large-inner-diameter LC column (Onyx™ Monolithic C18, LC Column 50 × 4.6 mm, 130 A) and desalting for 3 min with 2% mobile phase B at a flow rate of 200 μL/min. A series of gradients (flow rate, 200 μL/min) was used to elute the trapped peptides for separation and then they were further applied to a Mass Spectrometer. The complex peptide mixture was separated using a 120 min linear gradient, which consisted of the following steps: 2% B for 5 min, 3% to 35% B for 100 min, 35% to 95% B for 20 min, then maintaining isocratic conditions at 95% B for 10 min, and finally reverting to 2% B for 20 min of re-equilibration.

The mass spectrometer QE was operated in positive ion mode with the data-dependent acquisition strategy. Capillary temperature was set to 320 °C and spray voltage was set to 3.5 kV with sheath gas of 40, auxiliary gas of 20, S lens voltage was 55 volts and hearer temperature was 350 °C. One scan cycle included an MS1 scan (*m*/*z* 400–900) acquired at a resolution (full width at half-maximum) of 70,000 with an AGC target of 3 × 10^6^ ions over a maximum time of 100 ms, followed by ten MS2 scans with resolution of 17,500 at in source collision-induced dissociation activation mode to fragment the ten most abundant precursors found in the MS1 spectrum with a target setting of 1 × 10^5^ ions, an accumulation time of 50 ms, and an isolation window of 4 Da (m/z). Normalized HCD collision energy was set to 27%, and one microscan was acquired for each spectrum. The dynamic exclusion was enabled with the following settings: repeat count, 1; repeat duration, 30 s; exclusion list size, 500; exclusion duration, 20 s. In this study, two respective cell lysates of control groups (Mock, day 2 and day 4) and experimental groups (Infected, day 2 and day 4) were analyzed sequentially.

### 2.9. Proteomic Bioinformatics

To identify proteins in mock (control) and ZIKV infected FRPE cells, mass spectroscopy data was processed by Trans-proteomic Pipeline software [33,34] and Skyline daily v.3.5 software [35]. Fold change to mock samples were generated using intensity values from the MPPReport plugin in Skyline daily. To identify cellular processes that are affected during ZIKV infection, the list of proteins from mock vs. infected samples at 2 and 4 dpi were input into Ingenuity Pathway Analysis (QIAGEN, Hilden, Germany) and network diagrams were generated.

### 2.10. RNA Library Preparation and Sequencing

For RNA sequencing, FRPE cells were infected with ZIKV at an MOI of 1 and cell lysates were harvested at 2 and 3 dpi. ZIKV infections were done in biological triplicates for each time point. The uninfected controls were done in biological quadruplicates and pooled in duplicates for downstream analyses. Total RNA was isolated from the cells using an RNeasy Mini Kit (QIAGEN, Hilden, Germany). RNase-free DNase treatment was performed on the column to remove residual DNA. Library preparation and RNA sequencing analysis were done by Cedars-Sinai Genomics Core. Samples were assessed for concentration and quality using the Thermo Scientific NanoDrop 8000 Spectrophotometer (Thermo-Scientific, Waltham, MA, USA). RNA sequencing libraries were constructed using Illumina TruSeq RNA Sample Preparation Kit v2 (Illumina, San Diego, CA, USA), as per manufacturer’s instructions. Briefly, total RNA, with RNA integrity (RIN) scores of 9 or better (Agilent Bioanalyzer RNA 6000 Nano kit, Santa Clara, CA, USA) were used for RNA library preparation. The poly (A)+ RNA was then purified from one microgram of total RNA from each sample using oligo-dT attached magnetic beads with two rounds of purification. Subsequently, the poly (A)+ RNA was fragmented and primed for cDNA synthesis as per manufacturer’s recommendations. RNA adapters and barcodes were ligated to cDNA to allow for clonal amplification and multiplexing. Sequencing was done on an Illumina NextSeq 500 using 75 bp single-end sequencing kit to yield an average read depth of 28 million reads per sample with a minimum number of reads for a sample of at least 16 million reads. The sequencing data was deposited to the Gene Expression Omnibus (GEO) database system with the accession number GSE83900.

### 2.11. Analysis of RNA Sequencing Data

Raw reads obtained from RNA-sequencing were aligned to the human genome using STAR (version 2.5) [36] with a custom human GRCh38 transcriptome reference downloaded from GENCODE, which contains all protein-coding and non-coding RNA genes based on human GENCODE version 23 annotation [37].

Expression counts for each gene in all the samples were normalized by a modified trimmed mean of the M-values normalization method. The unsupervised principal component analysis (PCA) was performed with DESeq2 Bioconductor package version 1.10.1 in R version 3.2.2 [38]. Each gene was fitted into a negative binomial generalized linear model. The Wald test was applied to assess the differential expression between two sample groups by DESeq2. The Benjamini and Hochberg procedure [39] was applied to adjust for multiple hypothesis testing and differentially expressed gene candidates were selected with a false discovery rate of less than 0.10. For visualization of coordinated gene expression in the samples, a hierarchical clustering with the Pearson correlation distance matrix was performed with differentially expressed gene candidates using the Bioconductor gplots package (version 2.14.2) in R. Significantly expressed genes were assessed for pathway enrichment using DAVID release 6.7 [40] and Ingenuity Pathway Analysis [41] (QIAGEN, Hilden, Germany). The *significantly* enriched canonical pathways were defined as having a q-value of < 0.01.

### 2.12. Reverse Transcription-Quantitative PCR Analysis

Total RNA was extracted from mock and ZIKV-infected FRPE and iRSCs at the designated time points using an RNeasy Mini Kit (QIAGEN, Hilden, Germany). After treatment with RNase-free DNase, 1 μg of RNA was reverse-transcribed into cDNA using random hexamer primers and the SuperScript III Reverse Transcriptase Kit (Thermo-Scientific, Waltham, MA, USA), as recommended by the manufacturer. The following conditions were used for cDNA amplification: 65 °C for 5 min and 4 °C for 1 min, followed by 55 °C for 60 min and 72 °C for 15 min. Quantitative real-time PCR was carried out using Platinum SYBR Green qPCR SuperMix-UDG with ROX Kit (Thermo-Scientific, Waltham, MA, USA) by the QuantStudio™ 12K Flex Real-Time PCR System (Thermo-Scientific, Waltham, MA, USA). The relative concentration of each transcript was calculated using 2^−ΔCT^ method using Glyceraldehyde 3-phosphate dehydrogenase (GAPDH) threshold cycle (C_T_) values for normalization. The qPCR primer pairs for the mRNA transcript targets are provided in Table S1. The following conditions were used for transcript amplification: 50 °C for 2 min and 95 °C for 2 min, followed by 40 cycles of 95 °C for 15 s and 60 °C for 1 min.

### 2.13. Antiviral Compound Analysis

A compound screen in a dose–response was performed in Vero cells. The cells were seeded at a cell density of 1 × 10^4^ cells per well in 96-well plates. After 16 h of plating, the cells were infected with ZIKV (MOI of 1). After 4 hpi, the cells were treated with test compounds, ribavirin (RBV), 6-azauridine (6-AZA), glycyrrhizin (GLY), cyclopentenyl cytosine (CPEC), mycophenolic acid (MPA), sodium cholate hydrate (SCH), benzalkonium chloride (BZK) and curcumin (CRM) at the following concentrations of 1 μM, 5 μM, and 10 μM in triplicate. All compounds were acquired from Sigma Aldrich unless otherwise stated. Cells were infected and treated for 3 days, at which point viral plaques were counted. Similarly, FRPE cells were seeded at a cell density of 1 × 10^4^ cells per well in 96-well plates. After 16 h of plating, the cells were infected with ZIKV (MOI of 1) and immediately following infection, test compounds were added at the following concentrations of 1 μM, 5 μM and 10 μM in triplicate. Cells were infected and treated for 3 days, at which point cell viability and apoptosis was measured, and the antiviral activity was measured by immunostaining of viral antigen. The mock, vehicle (ZIKV), and infected plus compound treated samples were immunostained, as mentioned above, and images were analyzed using ImageXpressMICRO (Molecular Devices, San Jose, CA, USA) with multi-wavelength cell scoring macro software. For each well, the number of nuclei (DAPI) and cell count was determined for nine different locations within the well. Data acquisition was based on DAPI-normalization.

### 2.14. Western Blot Analysis

Cell lysates were resolved by SDS-PAGE using 4–15% pre-cast gradient gels (Bio-Rad, Hercules, CA, USA) before using the Trans-Blot turbo transfer system (Bio-Rad, Hercules, CA, USA) to transfer to a 0.2 µm PVDF membrane. Subsequently, the membranes were blocked with 5% skim milk and 0.2% Tween-20 in PBS at room temperature for 1 h. The membrane was then probed with rabbit monoclonal antibodies STAT1 and beta-actin (Cell Signaling Technology, Danvers, MA, USA). Stabilized Peroxidase Conjugated Goat anti-rabbit IgG (H + L) secondary antibody (Thermo-Scientific, Waltham, MA, USA) was added and detected by chemiluminescence (SuperSignal West Pico Chemiluminescent Substrate kit, Thermo-Scientific, Waltham, MA, USA).

### 2.15. Immunocytochemistry

Mock and infected FRPE or iRSCs were fixed with ≥ 99.8% methanol (Sigma-Aldrich, St. Louis, MO, USA) or 4% paraformaldehyde (Electron Microscopy Sciences, Hatfield, PA, USA) for 20–30 min, then washed three times with 1× DPBS (Dulbecco’s Phosphate-Buffered Saline) (Corning, Manassas, VA, USA). The cells were permeabilized with 3% Bovine Serum Albumin (BSA) (Sigma Life Sciences, St. Louis, MO, USA) and 0.1% Triton-X 100 in 1× DPBS. For ZIKV immunostaining, fixed and permeabilized cells were incubated with mouse monoclonal antibody against the Flavivirus Envelope protein at a 1:200 dilution for up to 6 h or overnight incubation at 4 °C. The secondary antibody was added at 1:1000 dilutions and incubated for 1 h at room temperature. For staining of RPE markers, fixed cells were incubated overnight at 4 °C with anti-bestrophin (BEST-1) mouse monoclonal primary antibody (EMD Millipore, Billerica, MA, USA), anti-retinal pigment epithelium 65 (RPE 65) monoclonal primary antibody (EMD Millipore, Billerica, MA, USA), and anti-tight junction protein 1 (zona occludens 1) (TJP1/ZO-1) rabbit polyclonal antibody (Thermo Fisher Scientific, Waltham, MA, USA) at a dilution of 1:200. The secondary antibodies, goat anti-mouse polyclonal antibody (Alexa Fluor 594) (Thermo Fisher Scientific, Waltham, MA, USA) and goat anti-rabbit polyclonal antibody (Alexa Fluor 568) (Thermo Fisher Scientific, Waltham, MA, USA) were added at a dilution of 1:1000, and were incubated for an hour at room temperature. For immunohistochemistry analysis of retinal organoids, sections were cut with cryostat (Leica), washed 3 times with 1× DPBS, blocked with 2% BSA, 5% normal goat serum (Cell Signaling Technology, Danvers, MA, USA), or 5% normal donkey serum (Jackson ImmunoResearch Laboratories, West Grove, PA, USA), and then incubated overnight with each primary antibody. The sections were incubated with secondary antibodies for one hour at room temperature in the same blocking buffer. In between antibody changes, cells were washed 3 times with 1× DPBS and left on the rocker for 5 min for each wash. Nuclei were stained with DAPI (4′,6-Diamidino-2-Phenylindole, Dihydrochloride) (Thermo Fisher Scientific, Waltham, MA, USA) at a dilution of 1:5000. Images were acquired using a Nikon Eclipse Ti Immunofluorescence Microscope with Nikon Intenselight C-HGFI, and LSM 700 confocal microscope with Zeiss software program.

### 2.16. Statistical Analysis

Three independent biological replicates were performed for each of the represented experiments in the study. The error bars in the graph reflects the standard deviation. *p*-values were determined by a two-tailed Student’s t-test and two-way ANOVA followed by Bonferroni test for multiple comparisons using GraphPad Prism (7.04). The significance was reported if the *p* value was *p* < 0.05 (*); *p* < 0.001 (**); and *p* < 0.0001 (***).

## 3. Results

### 3.1. Fetal Retinal Pigment Epithelial Cells Are Susceptible to ZIKV Infection

**Figure 1:**
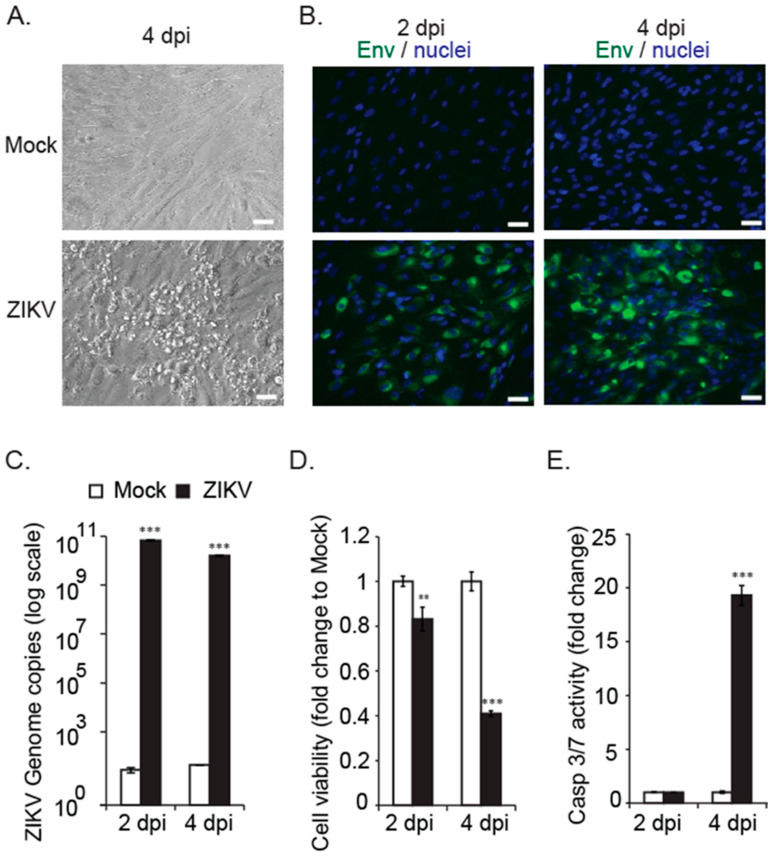
Fetal RPE cells are susceptible to ZIKV infection. (**A**) Bright field images of uninfected (mock) and ZIKV infected FRPE cells at 4 days post-infection (dpi). (**B**) Immunocytochemistry analysis of mock and ZIKV infected FRPE cells at 2 and 4 dpi. Flavivirus envelope protein is shown in green and nuclei in blue. Scale bar: 25 µm. (**C**) Graph shows the ZIKV genome copies (log scale) of mock and ZIKV infected cells at the indicated time points. (**D**) Bar graph shows reduced cell viability of ZIKV infected cells. (**E**) Caspase 3/7 activity measured for mock and ZIKV-infected cells at 2 and 4 dpi. Student t-test was performed for statistical significance, where the *p* value was *p* < 0.001 (**); and *p* < 0.0001 (***). Representative data from three independent experiments are shown.

To investigate the pathophysiology of ZIKV congenital eye infection, we employed an in vitro cell culture model system using human fetal retinal pigment epithelial cells from second- trimester of pregnancy to study their response to infection and to evaluate changes during ocular cell injury. For this study, we utilized a contemporary clinical ZIKV strain (PRVABC59) isolated in Puerto Rico during the 2015/2016 epidemic (Petersen et al., 2016). Fetal RPE cells were verified by the presence of retinal pigment epithelium-specific markers RPE65 and bestrophin (BEST1) (Figure S1A). FRPE cells did not form well-defined tight junctions as observed for adult RPE cells (Figure S1A). The FRPE cells expressed several flaviviral cell entry receptors GRP76, SDC2, HSP90AB1, TYRO3, AXL, and MERTK (Figure S1B). To understand the ZIKV cytopathic effect (CPE) in human ocular cells, FRPE cells were infected with a multiplicity of infection (MOI) of 0.1, as previously described [24,32]. FRPE cells showed active viral infection as evident by the presence of viral plaques on the cell monolayer (Figure 1A). Infected cells exhibited a distinctive raised pleomorphic phenotype and were detached from the cell’s monolayer (Figure 1A). ZIKV infection was confirmed by immunocytochemistry analysis of mock and infected FRPE cells using an antibody against the flavivirus envelope (Env) structural protein (Figure 1B). ZIKV genome replication was verified by RT-qPCR at 2 and 4 days post infection (dpi) (Figure 1C). To further characterize the cellular response to ZIKV infection, we examined cell viability. ZIKV infection of FRPE cells decreased cell viability at 2 and 4 dpi (20% vs. 60%) as compared to mock infected cells, which exhibited 100% viability (Figure 1D). We then evaluated the apoptotic effects of ZIKV on infected cells by measuring Caspase 3/7 activity. An increase in Caspase 3/7 activity was detected in ZIKV-infected cells at 4 dpi, suggesting the induction of programmed cell death (Figure 1E). FRPE cells were subjected to Annexin V staining to examine early apoptotic events, as defined by viral plaques. At 3 dpi, FRPE cells showed double staining for Annexin V and propidium iodide (PI), while mock-infected cells showed little or no measurable apoptosis (Figure S2). Overall, our data shows that the ZIKV-infected FRPE cells had increased caspase 3/7 activity and reduced cell viability due to robust viral infection.

### 3.2. Global Transcriptomics and Proteomics Analyses of ZIKV-Infected Fetal RPE Cells

**Figure 2:**
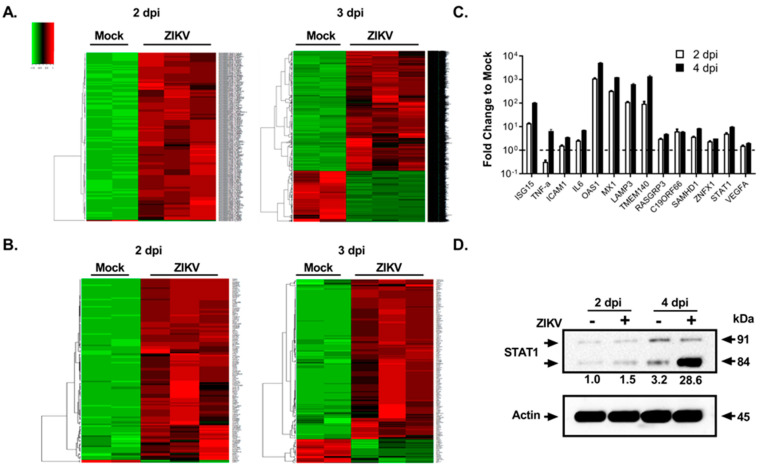
ZIKV dysregulates host cellular processes and activates innate immune and inflammatory responses in fetal RPE cells. (**A**) Heat map of differentially expressed genes at 2 dpi (2040 genes) and 3 dpi (7951 genes) [upregulated (red); downregulated (red)]. 2-way supervised Hierarchical Clustering was performed using unique genes identified for the combined dataset. (**B**) Heat maps show the differential expression of known canonical ISGs in mock and ZIKV infected cells at 2 dpi (128 genes) and 3 dpi (184 genes). (**C**) RT-qPCR validation of selected inflammatory and IFN-stimulated genes from (**B**) in ZIKV infected FRPE cells at 2 and 4 dpi. The fold change to mock is shown. The dash line represents the mock value at 1 where the fold change was taken for the specified time points. (**D**) Western blot showing the induction of STAT1 after ZIKV infection at 2 and 4 dpi. Note that higher levels of STAT1 beta subunit (84 kDa) are present in infected cells. Actin was included as a loading control. Actin normalized density of bands compared to that of 2 dpi mock is provided for 84 kDa beta STAT1 isoform. ZIKV infections were done in biological triplicates for each time point. The uninfected controls were done in biological quadruplicates and pooled in duplicate for downstream analyses.

**Figure 3:**
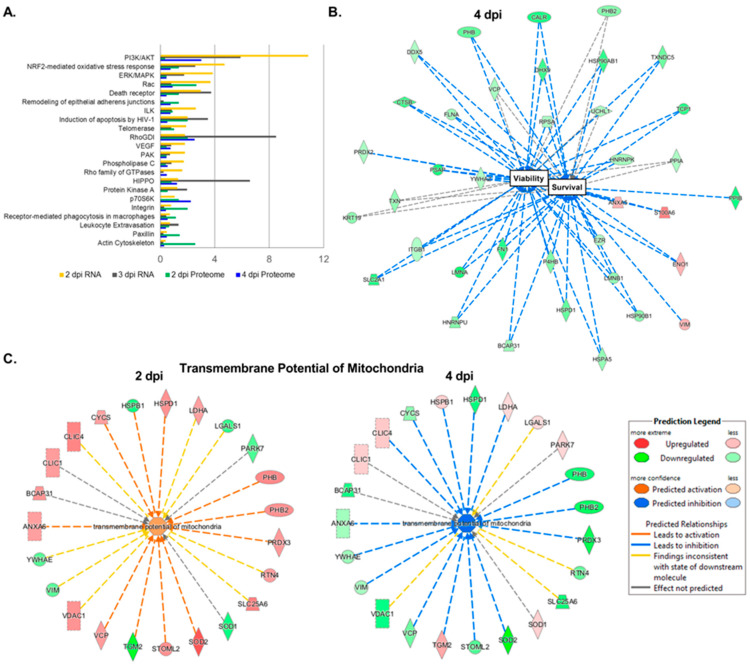
Proteomics analyses of human fetal RPE cells infected with ZIKV. (**A**) Graph shows key deregulated signaling pathways identified in ZIKV-infected FRPE cells through comprehensive proteomics and transcriptomics analyses. (**B**,**C**) Interaction networks of dysregulated factors involved in cell survival (**B**) and mitochondrial transmembrane potential (**C**) during ZIKV infection [upregulated (red); downregulated (green)]. For proteomics analyses, biological quadruplicates were performed and then samples were pooled in duplicate for mass spectrometry.

We then wanted to see the effects of ZIKV infection on FRPE cells at the molecular level by transcriptomics and proteomics analyses. RNA sequencing analysis of polyadenylated transcripts revealed that at 2 dpi, there were 2040 differentially regulated genes upon ZIKV infection, with a *p*-value less than 0.05 (Figure 2A and Table S2). At 3 dpi, there were 7951 differentially regulated genes after viral infection (Figure 2A and Table S3). Figure 2B provides a heat map of differentially expressed canonical interferon stimulated genes (ISGs) at 2 dpi (128 genes) and 3 dpi (184 genes). RT-qPCR validation of selected ISGs showed that ISG15, LAMP3, SAMHD1, RASGRP3, and C19ORF66 were induced in infected FRPE cells at both 2 and 4 dpi (Figure 2C). Moreover, infected cells had induced expression of pro-inflammatory genes TNFα, IL-6, and ICAM1 (Figure 2C). In vivo mice studies have shown that ZIKV directly inhibits STAT2 during infection [42]. In human FRPE cells, key JAK-STAT pathway transcription factors were upregulated during infection, including IFNAR1, IFNAR2, and STAT1 (Figure 2 and Tables S2 and S3). In addition, Western blot analysis showed the induction of total STAT1 protein in ZIKV infected cells at 2 and 4 dpi (Figure 2D). The upregulation of STAT1 suggests that the host cells are activating type I IFN antiviral response. Analysis of gene ontology categories and functional pathways showed the activation of various genes involved in the inflammasome pathway (NLRP1, NLRP3, ASC, AIM2, and NLRC4), the IL-17 signaling pathway, and the VEGFA signaling pathway, which is part of tissue healing and repair processes (Figure S3 and Tables S2 and S3). Infected FRPE cells showed increased levels of VEGFA as compared to mock cells (Figure 2C). Proteomics analyses identified 492 deregulated proteins (*p*-value less than 0.05) involved in diverse cellular processes, which included cell growth and proliferation, cell survival, metabolism, transcription, and protein synthesis (Table S4). The top survival pathways identified in both transcriptomics and proteomics analyses were PI3/AKT and ERK/MAPK (Figure 3A). Moreover, several proteins involved in cell survival (DDX5 and PHB), phagocytosis (TXNDC5), particle internalization (RPSA), and oxidative stress response (PRDX2 and TCP1) were differentially regulated (Figure 3B). Proteins involved in controlling mitochondrial membrane potential (CLIC1, CLIC4, and LDH4) were deregulated at both 2 and 4 dpi (Figure 3C). The Rac signaling pathway was one of many top hits in the proteomics common pathway analysis (Figure 3A). Overall, our results indicate that fetal RPE cells respond to ZIKV infection by activating innate immune, inflammatory, and stress response pathways.

### 3.3. Drug Treatment to Reduce ZIKV-Mediated Ocular Cell Death

**Figure 4:**
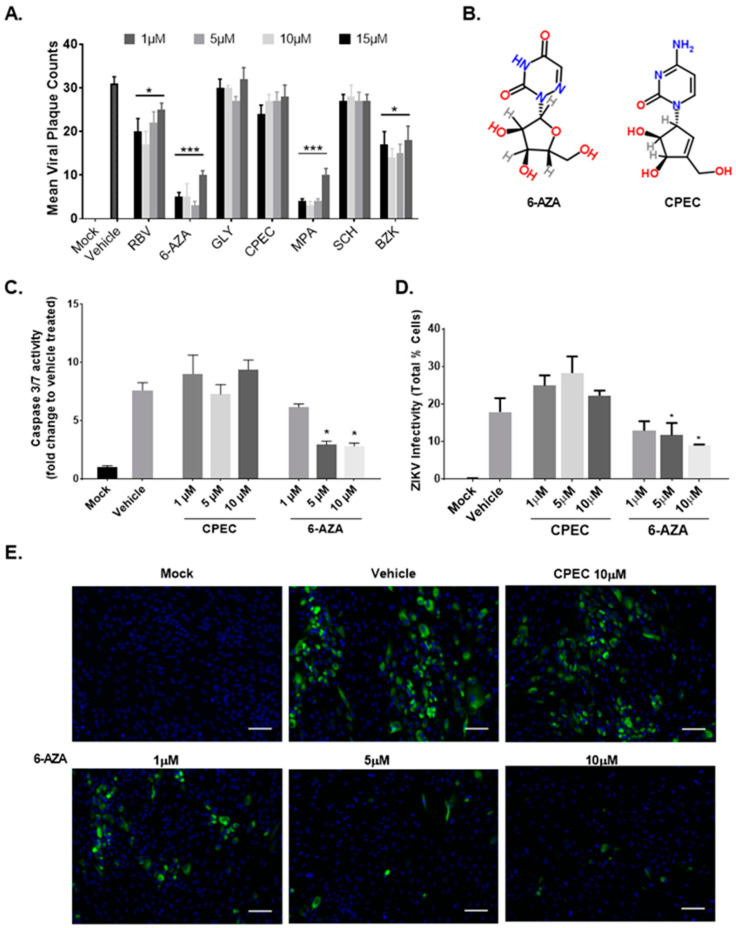
6-Azauridine inhibits ZIKV replication in FRPE cells. (**A**) Mean Zika viral plaque counts after an antiviral chemical compound screen in Vero cells. RBV (ribavirin); 6-AZA (6-Azauridine); GLY (Glycyrrhizin); CPEC (cyclopentenyl cytosine); MPA (mycophenolic acid); SCH (sodium cholate hydrate); BZK (benzalkonium chloride). Two-way ANOVA followed by Bonferroni test was performed. (**B**) Chemical compound structures of 6-AZA and CPEC. (**C**) Caspase 3/7 activity measured for mock (control), ZIKV infected FRPE cells (vehicle treated) (MOI of 1), and infected and treated FRPE cells with CPEC and 6-AZA at 1, 5, and 10 μM concentrations. (**D**) The percentage of ZIKV infected FRPE cells (% cells) as measured by immunofluorescence assay for detection of ZIKV Env. (**E**) Immunofluorescence images of mock, vehicle, and ZIKV infected and drug treated cells. Treatment with 6-AZA shows a dose dependent anti-viral effect in FRPE cells. Scale bar: 100 μm. Student t-test was performed for statistical significance if the *p*-value was *p* < 0.05 (*); *p* < 0.0001 (***). Representative data from three independent experiments are shown.

In order to identify an anti-ZIKV agent that can limit viral replication and cell injury, we first performed a small screen using selected compounds in Vero cells infected with ZIKV (Figure 4A), which have been previously shown to have anti-flaviviral activity. From this screen, we identified a nucleoside analogue 6-Azauridine (6-AZA) (Figure 4B) as a potent inhibitor of ZIKV. We then tested 6-AZA’s antiviral activity on infected fetal RPE cells. The drug compound cyclopentenyl cytosine (CPEC) has also been shown to have a broad spectrum anti-viral effect on West Nile virus [43], but it did not inhibit ZIKV replication in Vero cells (Figure 4A). To determine the effects of each compound on ZIKV-mediated apoptotic cell death of FRPE, we measured caspase 3/7 activity after ZIKV infection and drug treatment (1 μM, 5 μM, and 10 μM) at 3 days post-infection (hpi). 6-AZA treatment significantly reduced caspase 3/7 activity at 5 and 10 μM concentrations (Figure 4C), which correlated with the significant decrease in ZIKV infection (IC_50_ of 5 µM) (Figure 4D). CPEC had similar caspase 3/7 activity as the ZIKV-only infected group (vehicle control) (Figure 4C). Immunofluorescence assay confirmed the anti-viral activity of 6-AZA in FRPE cells by showing significantly reduced viral foci formation at 5 and 10 μM, as compared to the vehicle control (Figure 4E). There was no change observed in ZIKV replication in CPEC treated cells, which had similar levels of infected cell foci as the vehicle control (Figure 4E). Overall, 6-AZA showed an anti-ZIKV effect with a reduced apoptotic cellular response in FRPE cells, which can be further developed as a therapy against ZIKV infection.

### 3.4. Human iPSC-Derived Retinal Stem Cells (iRSCs) Are Susceptible to ZIKV Infection

**Figure 5:**
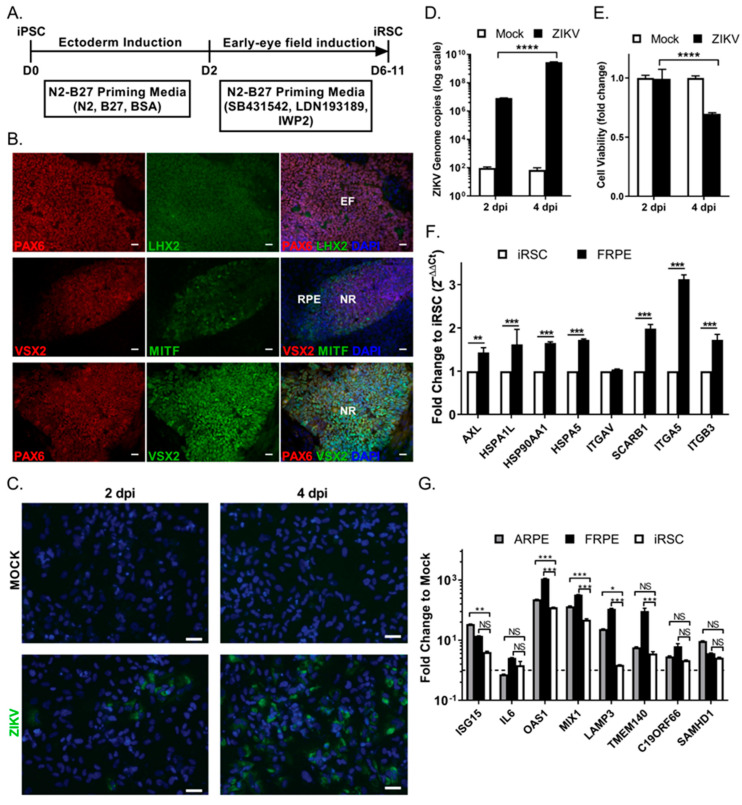
iPSC-derived retinal stem cells show reduced susceptibility to ZIKV infection (**A**) Schematic diagram showing the experimental outline used for the differentiation of human derived iPSCs into iRSCs. (**B**) Differentiated iRSCs show the expression of early eye stem cell transcription factors PAX6, LHX2, VSX2/CHX10 (neural retina; NR) and MITF (RPE) at day 11. Nuclei stained with DAPI. Scale bar: 30 μm. EF: eye field. (**C**) Immunofluorescence images of mock and ZIKV-infected iRSCs (viral Env protein, green) at 2 (left panels) and 4 (right panels) dpi. Nuclei stained with DAPI (blue). Scale bar: 25 μm. (**D**) Graph shows the quantification of ZIKV genome copies in mock and ZIKV infected iRSCs at 2 and 4 dpi. (**E**) Viability of uninfected and infected iRSCs at 2 and 4 dpi. The fold change to mock is shown. (**F**) Fold change in flaviviral entry receptor expression in fetal RPE cells relative to iRSCs. RT-qPCR Ct cycles for each gene were normalized to housekeeping gene GAPDH. The calculated 2^−ΔΔCt^ values are presented in the bar graph as fold change. Student t-test was performed (**G**) Comparative analysis of inflammatory and innate immune genes induced by ZIKV infection of fetal RPE (FRPE), adult RPE (ARPE), and iRSCs. Two-way ANOVA followed by Bonferroni test was performed for statistical significance if the *p* value was *p* < 0.05 (*); *p* < 0.001 (**); *p* < 0.0001 (***) and *p* < 0.00001 (****), and ns, no significance. Representative data from three independent experiments are shown.

We then evaluated the susceptibility of human iPSC-derived retinal stem cells (iRSCs), also known as eye field stem cells (EFSCs), to ZIKV infection. RSCs represent the multi-potent retinal stem/progenitor cell population present in the first trimester of the developing human eye. RSCs are able to differentiate into RPE cells, photoreceptors, retinal ganglion cells, and corneal endothelial cells. We previously reported the differentiation of iRSCs from human iPSCs and their detailed cellular and molecular characterization [30]. The iPSCs were directionally induced to form retinal stem cells (day 6–11) under chemically defined conditions, as shown in Figure 5A. The expression of retinal transcription factors PAX6, LHX2, MITF, and VSX2 (CHX10) were confirmed by immunofluorescence of iRSCs (Figure 5B). iRSCs were then infected with ZIKV at an MOI of 0.1. We found that iRSCs were susceptible to ZIKV infection, as shown by the presence of viral Env protein (green) in the cytoplasm of infected cells at 2 and 4 dpi (Figure 5C). iRSCs showed increased infectivity at 4 dpi, as reflected by ZIKV genome copies (Figure 5D). iRSC viability was affected by 4 dpi with a 35% reduction in infected cells as compared to mock cells (Figure 5E). iRSCs were less susceptible to ZIKV infection, as iRSCs had 4 log lower levels of viral genome copies as compared to that of FRPE cells at 2 dpi under similar infection conditions (Figure 1C and Figure 5D). The uninfected iRSCs showed reduced expression of selected flaviviral entry receptors AXL, heat shock proteins (HSP90AA1, and HSPA5), and integrins (ITGA3, and ITGA5) relative to FRPE cells (Figure 5F and Figure S1B). Differential receptor usage may partially explain the higher susceptibility of FRPE cells to ZIKV infection. It is possible that other intrinsic factors could contribute to the differential susceptibility. Moreover, ZIKV infected iRSCs had significantly reduced expression of many innate immune genes such as OAS1, MX1, LAMP3 and TMEM140, compared to infected fetal and adult RPE cells (Figure 5G). Overall, ZIKV-infected iRSCs showed a delay in viral infection, with a significant decrease in cell viability, and increased ZIKV genome copy number by 4 dpi.

### 3.5. ZIKV Infects hiPSC-Derived 3-Dimensional Retinal Organoids and Dysregulates the Differentiation Program

**Figure 6:**
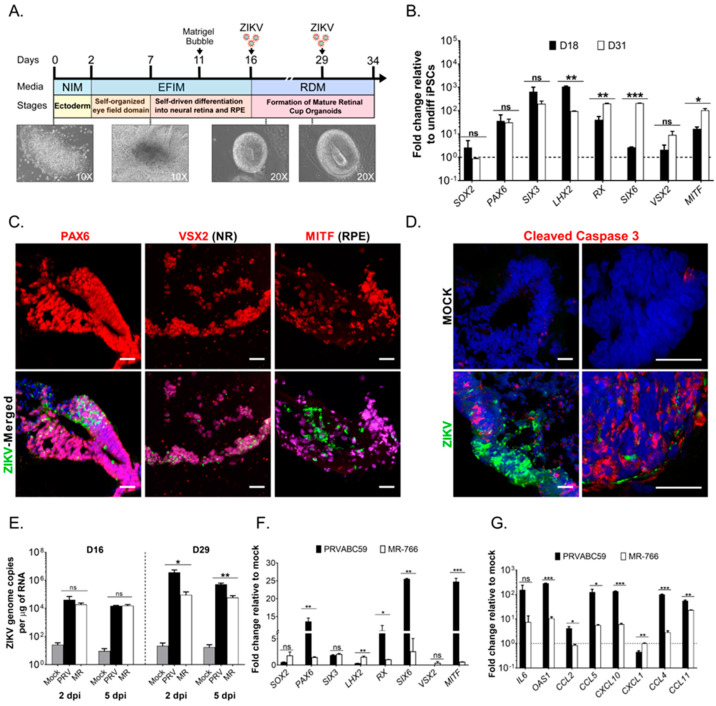
ZIKV infects the neural retina and RPE of human iPSC-derived Retinal Organoids. (**A**) Schematic timeline showing key differentiation stages of retinal organoids with stage-specific bright-field images. Organoids were infected with ZIKV on days 16 or 29 post-differentiation. (**B**) RT-qPCR analysis of early eye- and retinal-specific markers. Graph shows the fold change of each transcription factor relative to undifferentiated hiPSCs at days 18 and 31 post-differentiation. (**C**) Immunofluorescence images of mature retinal organoids infected on day 29 post-differentiation with ZIKV PRVABC59. Images show that PAX6, MITF (RPE), and VSX2 (neural retina, NR) positive cells (red) are infected with ZIKV (green) at 5 days post-infection (dpi). (**D**) Cleaved Caspase-3 (red) positive cells in mock and ZIKV-infected organoids at 5 dpi. Left panels (20×) and right panels (100×). Scale Bar: 25 µm. (**E**) Replication kinetics of ZIKV PRVABC59 and MR-766 of infected retinal organoids (day 16 and day 29 post-differentiation) at 2 and 5 dpi. Two-way ANOVA followed by Bonferroni test was performed. (**F**) RT-qPCR analysis of differentially expressed retinal markers in retinal organoids (day 16) after PRVABC59 and MR-766 infection (5 dpi). The fold change to uninfected (mock) organoids is shown. (**G**) Fold change in the expression levels of IL6, OAS1, and various chemokines at 5 dpi. Students t-test, where *p*-value <0.05 (*), *p* < 0.001 (**), and *p* < 0.0001 (***), and ns, no significance. Data are representative of three-independent experiments.

To understand the effect of ZIKV on developing neural retina and RPE progenitor cells, we established three-dimensional (3-D) retinal organoids using the human NHDF iPSC line (Figure 6). Organoids are self-organized tissues, which can closely reflect the complexity of an organ, thus provide deeper insight to understand human biological processes. Moreover, organoid’s unique potential to be used in a variety of functional and therapeutic studies with robust outcomes makes them an attractive model for studying viral replication and pathogenesis mechanisms. Along with the substantial progresses in generating retinal organoids via hiPSC differentiation, new approaches of specific retinal organoids have been developed through various methods to recapitulate different eye organ systems, including embryoid body-based retinal Cups [31], embryoid body-based bipotent retinal progenitors [44], cyst-based optic cup [45], adherent culture-based neural retina induction [46], and direct 3D induction to retinal organoids [47]. Figure 6A shows a schematic of the differentiation program used to derive retinal organoids. We successfully generated retinal organoids at different stages of differentiation (day 16 to 34). Retinal organoids were further characterized by their morphology and the molecular signature profile of different transcription factors involved in neuroectoderm and early retina formation (Figure 6A,B). Day 31 retinal organoids showed a higher expression of retinal markers such as SIX6 (ectoderm differentiation and eye development), RX (retinal cell fate determination), VSX2 (neural retina (NR) specification), and MITF (RPE cell specification) as compared to day 18 retinal organoids, confirming the maturation and differentiation into different retinal domains (Figure 5B). Using this system, we evaluated the susceptibility of retinal organoids to the Asian genotype ZIKV strain, PRVABC59. We found that retinal progenitor cells (PAX6 positive), RPE cells (MITF positive), and the neural retinal cells (VSX2 positive) were all susceptible to ZIKV infection (Figure 6C). The control isotype antibodies did not show any background staining (Figure S4A). PRVABC59 infection caused Caspase 3-dependent apoptotic cell death in retinal organoids (Figure 6D). We observed that both infected (ZIKV antigen positive) and uninfected nearby cells underwent apoptosis in the focal lesion, which may be mediated by paracrine effect of interferons released from infected cells. To further evaluate the replication kinetics of different ZIKV strains, we compared the infectivity rate of the low-pathogenic African genotype strain (MR-766) and the virulent Asian genotype (PRVABC59) strain, on both immature (day 16) and mature (day 29) retinal organoids. We observed that retinal organoids supported the infection of both viral strains (Figure 6E). However, PRVABC59 replicated at higher levels in mature RC organoids (day 29). In an independent experiment, we observed that PRVABC59 replicated at higher levels in fetal RPE cells (Figure S4B). Subsequently, we evaluated the effect of ZIKV infection on the retinal differentiation program. We focused on day 16 organoids since both ZIKV strains replicated at similar levels at this time point. We found that PRVABC59 caused significant dysregulation in the expression of retinal programming factors that include PAX6, RX (RAX), SIX6 and MITF (Figure 6F). In addition, PRVABC59 infection was associated with significantly increased expression levels of the inflammatory gene OAS-1 and chemokines CCL-2, CCL-4, CCL-5, and CCL-11 and CXCL10 (Figure 6G). These results suggest PRVABC59 elicits a strong pro-inflammatory response in different retinal cell types comprising retinal organoids, which may contribute to ocular cell injury. In conclusion, neural retina and RPE cells comprising human iPSC-derived retinal organoids support ZIKV infection and replication. Furthermore, the pathogenic Asian genotype strain had a significant impact on retinal organoids by dysregulating the optimal balance of the retinal differentiation program, which may be a result of the induction of inflammatory and apoptotic responses in the differentiating retinal cells.

## 4. Discussion

The severity and location of ocular cell injury caused by ZIKV contributes to different levels of visual impairment in children [5]. Our studies provide insight on the initial cellular response to ZIKV infection by examining cells that comprise different stages of eye development, including iRSCs and retinal organoids, which are equivalent to cells or optic cup tissue found in the first trimester of development, and human fetal RPE cells, which constitute the cells of the retina at the second-trimester (~20 weeks) [48,49]. We first evaluated the permissiveness of FRPE cells to ZIKV infection. We found that FRPE cells are highly susceptible to ZIKV infection, further leading to increased apoptosis and reduced cell viability. Transcriptomics analyses of infected FRPE cells showed a type I interferon signature indicative of innate antiviral immune pathway activation. Furthermore, infected FRPE cells showed activation of inflammasome and IL-17 signaling pathways. These pathways can amplify the inflammatory state by recruiting first-line defense cells, such as neutrophils, macrophages, and NK cells to the infected site in vivo. Furthermore, proteomics analyses revealed the dysregulation of factors involved in cell survival, such as the PI3K/AKT signaling pathway. This data set will serve as a resource to the scientific community to further dissect the pathobiology of ZIKV-mediated eye and brain diseases. 

During development, retinal stem cells are precursor progenitor cells to RPE cells, retinal ganglion cells, corneal endothelial cells, and photoreceptors [30,31,50,51,52]. For this reason, iRSCs are a useful in vitro model system to decipher the pathobiology of ZIKV eye infection at the early stages of ocular development. In this study, we showed that retinal stem cells are susceptible to ZIKV infection, but provides a lower degree of infection as compared to its downstream lineage of fetal RPE cells. Moreover, similar to studies with iPSC-derived neuroprogenitor cells (NPCs; 24 days post-differentiation) [52,53], we observed increased cell death of iRSCs (6 days post-differentiation) during active ZIKV replication. These data suggest that the cells at the stem stage of differentiation could be relatively less permissive to ZIKV infection. Furthermore, analysis of flaviviral entry receptor expression showed that FRPEs had higher levels of AXL, heat shock proteins, and integrins as compared to iRSCs, which may explain the differential susceptibility to infection in these cell types. It is also possible that iRSCs lack other pertinent cellular factors critical for robust ZIKV replication. ZIKV infection may impact the development of multiple cell fates, further leading to the generation of aberrant non-functional cells and/or malformations of the eye in the developing fetus. Moreover, retinal cup cells, including neural retina are derived from retinal stem cells and if the iRSCs are damaged by ZIKV during the first trimester, then abnormal development of the retinal cup will ensue. 

To address this, we established 3-dimensional retinal organoids. Human iPSCs have been shown to differentiate into retinal cups that recapitulate retinal development observed in vivo [31,49]. After successfully deriving mature RC organoids, we used this model system to study the molecular mechanism of ZIKV-mediated cell injury. Studies have shown that ZIKV causes teratogenic effects using human NPCs and brain/cerebral/neural organoids [19,53,54,55,56]. In our study, we have shown that ZIKV causes molecular changes to the expression of retinal programming factors, which could lead to abnormal differentiation. We observed that MITF-positive RPE cell layer and internal VSX2 expressing neural retinal cells are permissive to ZIKV infection, suggesting a broader ZIKV tropism, which can result in retinal abnormalities in a developing eye. 

Previous studies have showed that ZIKV can halt the cell cycle and induce apoptotic cell death in human neuroprogenitors and brain organoids [52,56,57]. In our studies, ZIKV induced caspase 3-mediated apoptosis in FRPE cells and retinal organoids. These results suggest that the tissue injury caused by apoptotic cell death could be a major contributing factor to the eye pathology observed in the fetus and affected infants. Viral particles released by apoptotic bodies can be taken up by neighboring cells and macrophages in vivo and in turn facilitate viral spread [57]. There is also evidence for active ZIKV infection of macrophages in the placenta of pregnant mothers, which in turn can infect the developing fetus [58]. In addition, proteomics analyses of infected FRPE cells showed activation of proteins controlling mitochondrial membrane potential. This increased mitochondrial potential could be due to the infected cell undergoing active apoptotic processes. The tissue damage caused by other flaviviruses can also be attributed to the activation of programmed cell death. For example, platelets from Dengue-infected patients show characteristic apoptotic features, such as the induction of Caspase-3 and -9, as well as mitochondrial polarization [59].

A previous study describing the humoral response during ZIKV infection of pregnant women with neonatal microcephaly, showed that amniotic fluid had increased levels of VEGF, pro-inflammatory cytokines (IL-8 and IL-6), and chemokines (IP-10 and MCP-1), which may contribute to other fetal developmental abnormalities [60]. Consistent with this study, we observed that ZIKV induces the expression of VEGF and various chemokines (CXCL1 and CCL5) in fetal RPE cells. RPE cells require VEGF for the health of the endothelium and underlying blood vessels [61,62]. VEGF binds to the receptor Flt1 and KDR on endothelial cells and can initiate different intracellular signaling pathways, including PI3/AKT, FAK, PKC, and MAPK pathways to promote cell survival and angiogenesis [63]. VEGF has also been implicated in the pathogenesis of an important degenerative eye disease. In wet form of age-related macular degeneration (AMD) disease, overproduction of VEGF leads to abnormal growth of choriocapillaris [64]. Congenital eye lesions caused by ZIKV may resemble macular degeneration [5]. In vivo studies may provide insight on the role of VEGF during ZIKV-mediated eye disease.

Currently, there are no approved antiviral agents to treat and prevent ZIKV-mediated disease. In this study, we tested drug compounds previously shown to have antiviral effects on different flaviviral pathogens, including Dengue, Yellow Fever, and West Nile viruses [65]. Our in vitro studies show that treatment with 6-AZA blocked virus production and apoptosis in FRPE cells immediately after ZIKV infection. 6-AZA was recently shown to have anti-ZIKV activity [66,67]. In general, nucleoside analogues are incorporated into the newly synthesized viral genome, further causing deleterious mutations. Furthermore, the ZIKV polymerase NS5 may be a target for such nucleoside analogues. Recently, additional drug compounds with anti-ZIKV activity have been described [68,69,70]. Although vaccines are the best choice for preventing ZIKV infection in pregnant women, antiviral drugs can be used as a post-exposure therapeutic option. Moreover, hiPSC-RPE is increasingly used in the study of diseases affecting retina [71,72,73,74,75], which can be used for ZIKV infection studies [76] and further evaluating the safety and efficacy of the identified Zika antiviral compounds. In summary, this study provides a snapshot into ZIKV infection at the first and second-trimester of retinal development using biologically relevant in vitro ocular cell model systems. Our study presents a comprehensive assessment of pathways affected in fetal ocular cells by transcriptomics and proteomics analyses. The in vitro retinal organoid-ZIKV model system can be used to further investigate the role of viral virulence factors and host-genetic determinants contributing to the pathogenesis of congenital ZIKV eye disease.

## Data Availability

Not applicable.

## Supplementary Materials

The following supporting information may be downloaded at: https://www.mdpi.com/article/10.3390/v15010142/s1, Figure S1: Fetal and adult RPE cells express specific retinal pigment epithelial cell markers; Figure S2: ZIKV induces apoptotic cell death in fetal RPE cells; Figure S3: Transcriptome and pathway analyses of infected FRPE cells at 3 dpi; Figure S4: Analysis of Retinal organoids and ZIKV infectivity study; Table S1: List of RT-qPCR primers used in this study; Table S2: List of differentially expressed genes in ZIKV infected FRPE cells at 2 dpi as compared to mock; Table S3: List of differentially expressed genes in ZIKV infected FRPE cells at 3 dpi as compared to mock; Table S4: List of differentially expressed proteins in ZIKV infected FRPE cells at 2 and 4 dpi; Table S5: List of antibodies used in this study.

## References

[1] Jampol L.M., Goldstein D.A. (2016). Zika Virus Infection and the Eye. JAMA Ophthalmol..

[2] Brasil P., Pereira J.P., Moreira M.E., Ribeiro Nogueira R.M., Damasceno L., Wakimoto M., Rabello R.S., Valderramos S.G., Halai U.A., Salles T.S. (2016). Zika Virus Infection in Pregnant Women in Rio de Janeiro. New Engl. J. Med..

[3] Hamel R., Liegeois F., Wichit S., Pompon J., Diop F., Talignani L., Thomas F., Despres P., Yssel H., Misse D. (2016). Zika virus: Epidemiology, clinical features and host-virus interactions. Microbes Infect..

[4] Kim K., Shresta S. (2016). Neuroteratogenic Viruses and Lessons for Zika Virus Models. Trends Microbiol..

[5] De Paula Freitas B., de Oliveira Dias J.R., Prazeres J., Sacramento G.A., Ko A.I., Maia M., Belfort R. (2016). Ocular Findings in Infants with Microcephaly Associated With Presumed Zika Virus Congenital Infection in Salvador, Brazil. JAMA Ophthalmol..

[6] Miner J.J., Sene A., Richner J.M., Smith A.M., Santeford A., Ban N., Weger-Lucarelli J., Manzella F., Ruckert C., Govero J. (2016). Zika Virus Infection in Mice Causes Panuveitis with Shedding of Virus in Tears. Cell Rep..

[7] Dos Santos T., Rodriguez A., Almiron M., Sanhueza A., Ramon P., de Oliveira W.K., Coelho G.E., Badaro R., Cortez J., Ospina M. (2016). Zika Virus and the Guillain-Barre Syndrome—Case Series from Seven Countries. New Engl. J. Med..

[8] Lucchese G., Kanduc D. (2016). Zika virus and autoimmunity: From microcephaly to Guillain-Barre syndrome, and beyond. Autoimmun. Rev..

[9] Parra B., Lizarazo J., Jimenez-Arango J.A., Zea-Vera A.F., Gonzalez-Manrique G., Vargas J., Angarita J.A., Zuniga G., Lopez-Gonzalez R., Beltran C.L. (2016). Guillain-Barre Syndrome Associated with Zika Virus Infection in Colombia. New Engl. J. Med..

[10] Chen B.Y., Chang H.H., Chen S.T., Tsao Z.J., Yeh S.M., Wu C.Y., Lin D.P. (2009). Congenital eye malformations associated with extensive periocular neural crest apoptosis after influenza B virus infection during early embryogenesis. Mol. Vis..

[11] Graw J. (2003). The genetic and molecular basis of congenital eye defects. Nat. Rev. Genet..

[12] Lupo G., Andreazzoli M., Gestri G., Liu Y., He R.Q., Barsacchi G. (2000). Homeobox genes in the genetic control of eye development. Int. J. Dev. Biol..

[13] Ventura C.V., Maia M., Dias N., Ventura L.O., Belfort R. (2016). Zika: Neurological and ocular findings in infant without microcephaly. Lancet.

[14] Ventura C.V., Maia M., Travassos S.B., Martins T.T., Patriota F., Nunes M.E., Agra C., Torres V.L., van der Linden V., Ramos R.C. (2016). Risk Factors Associated With the Ophthalmoscopic Findings Identified in Infants With Presumed Zika Virus Congenital Infection. JAMA Ophthalmol..

[15] Vogel G. (2016). Experts fear Zika’s effects may be even worse than thought. Science.

[16] Mohr E.L., Block L.N., Newman C.M., Stewart L.M., Koenig M., Semler M., Breitbach M.E., Teixeira L.B.C., Zeng X., Weiler A.M. (2018). Ocular and uteroplacental pathology in a macaque pregnancy with congenital Zika virus infection. PLoS ONE.

[17] Rasmussen S.A., Jamieson D.J., Honein M.A., Petersen L.R. (2016). Zika Virus and Birth Defects—Reviewing the Evidence for Causality. N. Engl. J. Med..

[18] Ventura C.V., Ventura L.O. (2018). Ophthalmologic Manifestations Associated With Zika Virus Infection. Pediatrics.

[19] Cugola F.R., Fernandes I.R., Russo F.B., Freitas B.C., Dias J.L., Guimaraes K.P., Benazzato C., Almeida N., Pignatari G.C., Romero S. (2016). The Brazilian Zika virus strain causes birth defects in experimental models. Nature.

[20] Lazear H.M., Govero J., Smith A.M., Platt D.J., Fernandez E., Miner J.J., Diamond M.S. (2016). A Mouse Model of Zika Virus Pathogenesis. Cell Host Microbe.

[21] Li C., Xu D., Ye Q., Hong S., Jiang Y., Liu X., Zhang N., Shi L., Qin C.F., Xu Z. (2016). Zika Virus Disrupts Neural Progenitor Development and Leads to Microcephaly in Mice. Cell Stem Cell.

[22] Miner J.J., Cao B., Govero J., Smith A.M., Fernandez E., Cabrera O.H., Garber C., Noll M., Klein R.S., Noguchi K.K. (2016). Zika Virus Infection during Pregnancy in Mice Causes Placental Damage and Fetal Demise. Cell.

[23] Li Y., Shi S., Xia F., Shan C., Ha Y., Zou J., Adam A., Zhang M., Wang T., Liu H. (2021). Zika virus induces neuronal and vascular degeneration in developing mouse retina. Acta Neuropathol. Commun..

[24] Garcia G., Paul S., Beshara S., Ramanujan V.K., Ramaiah A., Nielsen-Saines K., Li M.M.H., French S.W., Morizono K., Kumar A. (2020). Hippo Signaling Pathway Has a Critical Role in Zika Virus Replication and in the Pathogenesis of Neuroinflammation. Am. J. Pathol..

[25] Martinez L.E., Garcia G., Contreras D., Gong D., Sun R., Arumugaswami V. (2020). Zika Virus Mucosal Infection Provides Protective Immunity. J. Virol..

[26] Miner J.J., Diamond M.S. (2017). Zika Virus Pathogenesis and Tissue Tropism. Cell Host Microbe.

[27] Maleski A.L.A., Rosa J.G.S., Bernardo J.T.G., Astray R.M., Walker C.I.B., Lopes-Ferreira M., Lima C. (2022). Recapitulation of Retinal Damage in Zebrafish Larvae Infected with Zika Virus. Cells.

[28] Salinas S., Erkilic N., Damodar K., Moles J.P., Fournier-Wirth C., Van de Perre P., Kalatzis V., Simonin Y. (2017). Zika Virus Efficiently Replicates in Human Retinal Epithelium and Disturbs Its Permeability. J. Virol..

[29] Singh P.K., Khatri I., Jha A., Pretto C.D., Spindler K.R., Arumugaswami V., Giri S., Kumar A., Bhasin M.K. (2018). Determination of system level alterations in host transcriptome due to Zika virus (ZIKV) Infection in retinal pigment epithelium. Sci. Rep..

[30] Zhao J.J., Afshari N.A. (2016). Generation of Human Corneal Endothelial Cells via In Vitro Ocular Lineage Restriction of Pluripotent Stem Cells. Investig. Ophthalmol. Vis. Sci..

[31] Zhong X., Gutierrez C., Xue T., Hampton C., Vergara M.N., Cao L.H., Peters A., Park T.S., Zambidis E.T., Meyer J.S. (2014). Generation of three-dimensional retinal tissue with functional photoreceptors from human iPSCs. Nat. Commun..

[32] Contreras D., Arumugaswami V. (2016). Zika Virus Infectious Cell Culture System and the In Vitro Prophylactic Effect of Interferons. J. Vis. Exp..

[33] Deutsch E.W., Mendoza L., Shteynberg D., Farrah T., Lam H., Tasman N., Sun Z., Nilsson E., Pratt B., Prazen B. (2010). A guided tour of the Trans-Proteomic Pipeline. Proteomics.

[34] Keller A., Eng J., Zhang N., Li X.J., Aebersold R. (2005). A uniform proteomics MS/MS analysis platform utilizing open XML file formats. Mol. Syst. Biol..

[35] MacLean B., Tomazela D.M., Shulman N., Chambers M., Finney G.L., Frewen B., Kern R., Tabb D.L., Liebler D.C., MacCoss M.J. (2010). Skyline: An open source document editor for creating and analyzing targeted proteomics experiments. Bioinformatics.

[36] Langmead B., Trapnell C., Pop M., Salzberg S.L. (2009). Ultrafast and memory-efficient alignment of short DNA sequences to the human genome. Genome Biol..

[37] Gencode. http://www.gencodegenes.org.

[38] Love M.I., Huber W., Anders S. (2014). Moderated estimation of fold change and dispersion for RNA-seq data with DESeq2. Genome Biol..

[39] Benjamini Y., Hochberg Y. (1995). Controling the false discovery rate: A practical and powerful approach to rapid testing. J. R. Stat. Soc..

[40] DAVID Bioinformatics Resources. https://david.ncifcrf.gov.

[41] QIAGEN Ingenuity Pathway Analysis (IPA). http://www.ingenuity.com/products/ipa.

[42] Grant A., Ponia S.S., Tripathi S., Balasubramaniam V., Miorin L., Sourisseau M., Schwarz M.C., Sanchez-Seco M.P., Evans M.J., Best S.M. (2016). Zika Virus Targets Human STAT2 to Inhibit Type I Interferon Signaling. Cell Host Microbe.

[43] Morrey J.D., Smee D.F., Sidwell R.W., Tseng C. (2002). Identification of active antiviral compounds against a New York isolate of West Nile virus. Antivir. Res..

[44] Shrestha R., Wen Y.T., Ding D.C., Tsai R.K. (2019). Aberrant hiPSCs-Derived from Human Keratinocytes Differentiates into 3D Retinal Organoids that Acquire Mature Photoreceptors. Cells.

[45] Lowe A., Harris R., Bhansali P., Cvekl A., Liu W. (2016). Intercellular Adhesion-Dependent Cell Survival and ROCK-Regulated Actomyosin-Driven Forces Mediate Self-Formation of a Retinal Organoid. Stem Cell Rep..

[46] Reichman S., Terray A., Slembrouck A., Nanteau C., Orieux G., Habeler W., Nandrot E.F., Sahel J.A., Monville C., Goureau O. (2014). From confluent human iPS cells to self-forming neural retina and retinal pigmented epithelium. Proc. Natl. Acad. Sci. USA.

[47] Zhang X., Jin Z.B. (2021). Directed Induction of Retinal Organoids from Human Pluripotent Stem Cells. J. Vis. Exp..

[48] Reh T.A., Fischer A.J. (2006). Retinal stem cells. Methods Enzymol..

[49] Volkner M., Zschatzsch M., Rostovskaya M., Overall R.W., Busskamp V., Anastassiadis K., Karl M.O. (2016). Retinal Organoids from Pluripotent Stem Cells Efficiently Recapitulate Retinogenesis. Stem Cell Rep..

[50] Parameswaran S., Balasubramanian S., Babai N., Qiu F., Eudy J.D., Thoreson W.B., Ahmad I. (2010). Induced pluripotent stem cells generate both retinal ganglion cells and photoreceptors: Therapeutic implications in degenerative changes in glaucoma and age-related macular degeneration. Stem Cells.

[51] Osakada F., Ikeda H., Mandai M., Wataya T., Watanabe K., Yoshimura N., Akaike A., Sasai Y., Takahashi M. (2008). Toward the generation of rod and cone photoreceptors from mouse, monkey and human embryonic stem cells. Nat. Biotechnol..

[52] Nakano T., Ando S., Takata N., Kawada M., Muguruma K., Sekiguchi K., Saito K., Yonemura S., Eiraku M., Sasai Y. (2012). Self-formation of optic cups and storable stratified neural retina from human ESCs. Cell Stem Cell.

[53] McGrath E.L., Rossi S.L., Gao J., Widen S.G., Grant A.C., Dunn T.J., Azar S.R., Roundy C.M., Xiong Y., Prusak D.J. (2017). Differential Responses of Human Fetal Brain Neural Stem Cells to Zika Virus Infection. Stem Cell Rep..

[54] Tang H., Hammack C., Ogden S.C., Wen Z., Qian X., Li Y., Yao B., Shin J., Zhang F., Lee E.M. (2016). Zika Virus Infects Human Cortical Neural Progenitors and Attenuates Their Growth. Cell Stem Cell.

[55] Souza B.S., Sampaio G.L., Pereira C.S., Campos G.S., Sardi S.I., Freitas L.A., Figueira C.P., Paredes B.D., Nonaka C.K., Azevedo C.M. (2016). Zika virus infection induces mitosis abnormalities and apoptotic cell death of human neural progenitor cells. Sci. Rep..

[56] Watanabe M., Buth J.E., Vishlaghi N., de la Torre-Ubieta L., Taxidis J., Khakh B.S., Coppola G., Pearson C.A., Yamauchi K., Gong D. (2017). Self-Organized Cerebral Organoids with Human-Specific Features Predict Effective Drugs to Combat Zika Virus Infection. Cell Rep..

[57] Roulston A., Marcellus R.C., Branton P.E. (1999). Viruses and apoptosis. Annu. Rev. Microbiol..

[58] Jurado K.A., Simoni M.K., Tang Z., Uraki R., Hwang J., Householder S., Wu M., Lindenbach B.D., Abrahams V.M., Guller S. (2016). Zika virus productively infects primary human placenta-specific macrophages. JCI Insight.

[59] Hottz E.D., Oliveira M.F., Nunes P.C., Nogueira R.M., Valls-de-Souza R., Da Poian A.T., Weyrich A.S., Zimmerman G.A., Bozza P.T., Bozza F.A. (2013). Dengue induces platelet activation, mitochondrial dysfunction and cell death through mechanisms that involve DC-SIGN and caspases. J. Thromb. Haemost..

[60] Ornelas A.M.M., Paula P., Silveira P.P., Melo F.O., Ferreira T.A., Oliveira-Szejnfeld P.S., Leal J.I., Amorim M.M.R., Hamilton S., Rawlinson W.D. (2016). Immune activation in amniotic fluid from Zika virus associated microcephaly. Ann. Neurol..

[61] Ford K.M., Saint-Geniez M., Walshe T., Zahr A., D’Amore P.A. (2011). Expression and role of VEGF in the adult retinal pigment epithelium. Investig. Ophthalmol. Vis. Sci..

[62] Nagineni C.N., Kommineni V.K., William A., Detrick B., Hooks J.J. (2012). Regulation of VEGF expression in human retinal cells by cytokines: Implications for the role of inflammation in age-related macular degeneration. J. Cell. Physiol..

[63] Eichmann A., Simons M. (2012). VEGF signaling inside vascular endothelial cells and beyond. Curr. Opin. Cell Biol..

[64] Kovach J.L., Schwartz S.G., Flynn H.W., Scott I.U. (2012). Anti-VEGF Treatment Strategies for Wet AMD. J. Ophthalmol..

[65] Crance J.M., Scaramozzino N., Jouan A., Garin D. (2003). Interferon, ribavirin, 6-azauridine and glycyrrhizin: Antiviral compounds active against pathogenic flaviviruses. Antivir. Res..

[66] Pascoalino B.S., Courtemanche G., Cordeiro M.T., Gil L.H., Freitas-Junior L. (2016). Zika antiviral chemotherapy: Identification of drugs and promising starting points for drug discovery from an FDA-approved library. F1000Research.

[67] Adcock R.S., Chu Y.K., Golden J.E., Chung D.H. (2017). Evaluation of anti-Zika virus activities of broad-spectrum antivirals and NIH clinical collection compounds using a cell-based, high-throughput screen assay. Antivir. Res..

[68] Xu M., Lee E.M., Wen Z., Cheng Y., Huang W.K., Qian X., Tcw J., Kouznetsova J., Ogden S.C., Hammack C. (2016). Identification of small-molecule inhibitors of Zika virus infection and induced neural cell death via a drug repurposing screen. Nat. Med..

[69] Micewicz E.D., Khachatoorian R., French S.W., Ruchala P. (2018). Identification of novel small-molecule inhibitors of Zika virus infection. Bioorg. Med. Chem. Lett..

[70] Baz M., Boivin G. (2019). Antiviral Agents in Development for Zika Virus Infections. Pharmaceuticals.

[71] Shrestha R., Wen Y.T., Tsai R.K. (2020). Effective Differentiation and Biological Characterization of Retinal Pigment Epithelium Derived from Human Induced Pluripotent Stem Cells. Curr. Eye Res..

[72] Osakada F., Jin Z.B., Hirami Y., Ikeda H., Danjyo T., Watanabe K., Sasai Y., Takahashi M. (2009). In vitro differentiation of retinal cells from human pluripotent stem cells by small-molecule induction. J. Cell Sci..

[73] Reichman S., Slembrouck A., Gagliardi G., Chaffiol A., Terray A., Nanteau C., Potey A., Belle M., Rabesandratana O., Duebel J. (2017). Generation of Storable Retinal Organoids and Retinal Pigmented Epithelium from Adherent Human iPS Cells in Xeno-Free and Feeder-Free Conditions. Stem Cells.

[74] Kamao H., Mandai M., Okamoto S., Sakai N., Suga A., Sugita S., Kiryu J., Takahashi M. (2014). Characterization of human induced pluripotent stem cell-derived retinal pigment epithelium cell sheets aiming for clinical application. Stem Cell Rep..

[75] Maeda T., Lee M.J., Palczewska G., Marsili S., Tesar P.J., Palczewski K., Takahashi M., Maeda A. (2013). Retinal pigmented epithelial cells obtained from human induced pluripotent stem cells possess functional visual cycle enzymes in vitro and in vivo. J. Biol. Chem..

[76] Simonin Y., Erkilic N., Damodar K., Clé M., Desmetz C., Bolloré K., Taleb M., Torriano S., Barthelemy J., Dubois G. (2019). Zika virus induces strong inflammatory responses and impairs homeostasis and function of the human retinal pigment epithelium. EBioMedicine.

