# Supplementary material for: Differential Susceptibility of Fetal Retinal Pigment Epithelial Cells, hiPSC- Retinal Stem Cells, and Retinal Organoids to Zika Virus Infection
Source: Viruses. 2023 Jan 1;15(1):142. doi: 10.3390/v15010142 (PMC9864143; doi:10.3390/v15010142)
Supplement: Supplementary file 1 [file viruses-15-00142-s001.zip › viruses-2066094-supplementary.pdf]

Supplemental File

Differential Susceptibility of Fetal Retinal Pigment Epithelial Cells, iPSC- Retinal Stem Cells, and Retinal Organoids to Zika Virus Infection

Running title: ZIKV infection of retinal cells and organoids

Deisy Contreras[1,2‡], Gustavo Garcia, Jr.[3‡], Melissa Kaye Jones[1,2], Laura E. Martinez[3], Akshaya Jayakarunakaran[3], Vineela Gangalapudi[4], Jie Tang[3,3], Ying Wu[5], Jiagang J. Zhao[5], Zhaohui Chen[2], Arunachalam Ramaiah[6], Irena Tsui[7], Ashok Kumar[8], Karin Nielsen-Saines[9], Shaomei Wang[1,2], Vaithilingaraja Arumugaswami[1,2,3,10*]

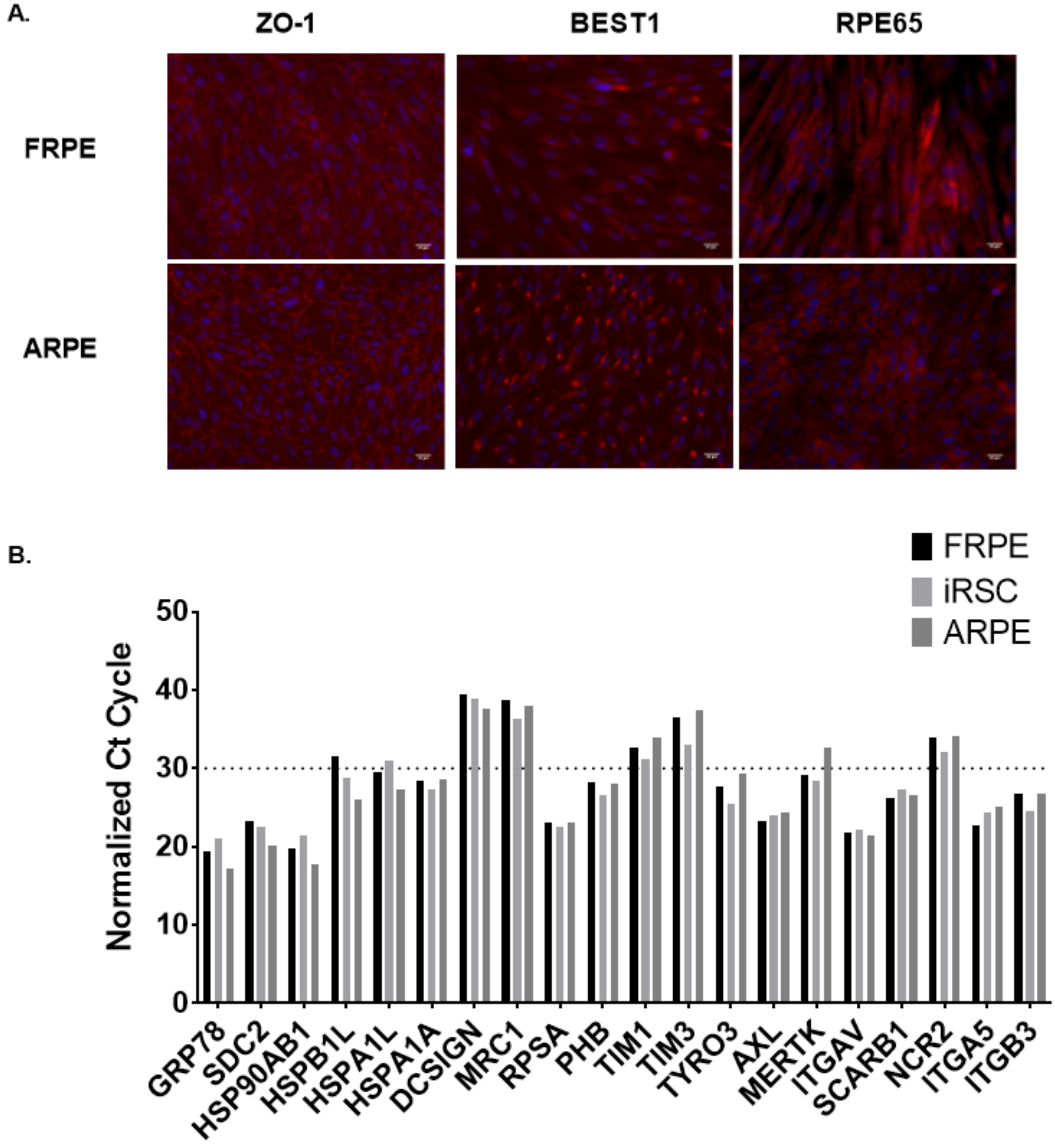

Supplemental Figure S1. Fetal and adult RPE cells express specific retinal pigment epithelial cell markers. Related to Figure 1. (A) Immunofluorescence images showing the expression of ZO-1 and RPE cell markers RPE65 and BEST1 (red) in fetal (FRPE) and adult RPE (ARPE) cells. Nuclei were stained with DAPI (blue). (B) The expression of various flaviviral entry receptors in FRPE, ARPE, and iRSCs. Provided are the RT-qPCR Ct cycle values normalized to GAPDH expression. Note the Ct values are inversely related to the expression level of each receptor. Representative data from three independent experiments are shown.

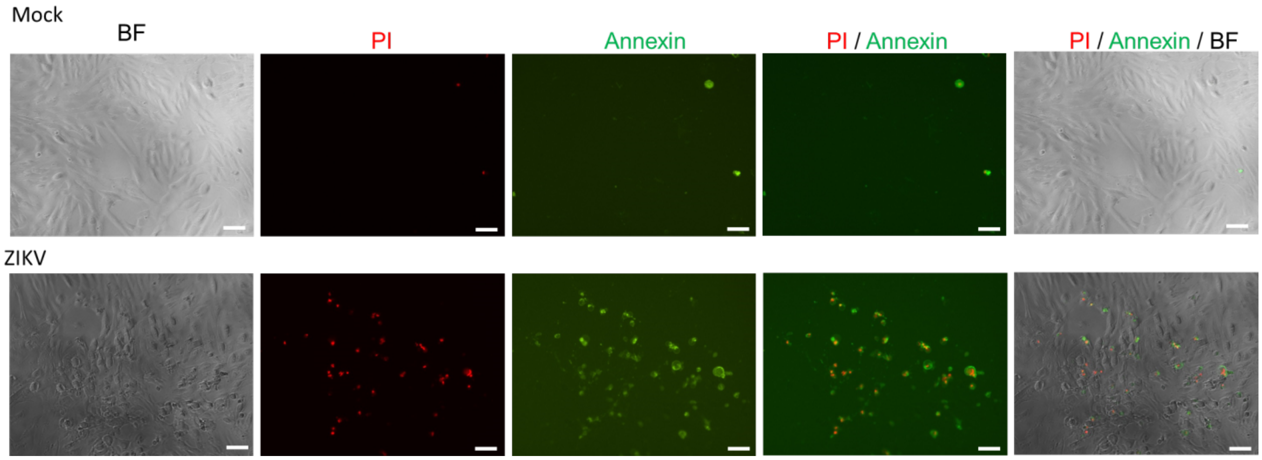

Supplemental Figure S2. ZIKV induces apoptotic cell death in fetal RPE cells. Related to Figure 1. Live cell images showing positive staining for Annexin V in mock and ZIKV infected FRPE cells at 3 dpi. Annexin V and/or Propidium Iodide (PI) were added to both mock and ZIKV infected cells for 15 minutes, as per manufacturer's instructions. Merged images show double positive staining of apoptotic and dead cells in viral plaques. Scale bar: 25 µm. Images were acquired using a Nikon Eclipse Ti Immunofluorescence Microscope with Nikon Intenselight C-HGFI. Representative data from three independent experiments are shown.

A.

B.

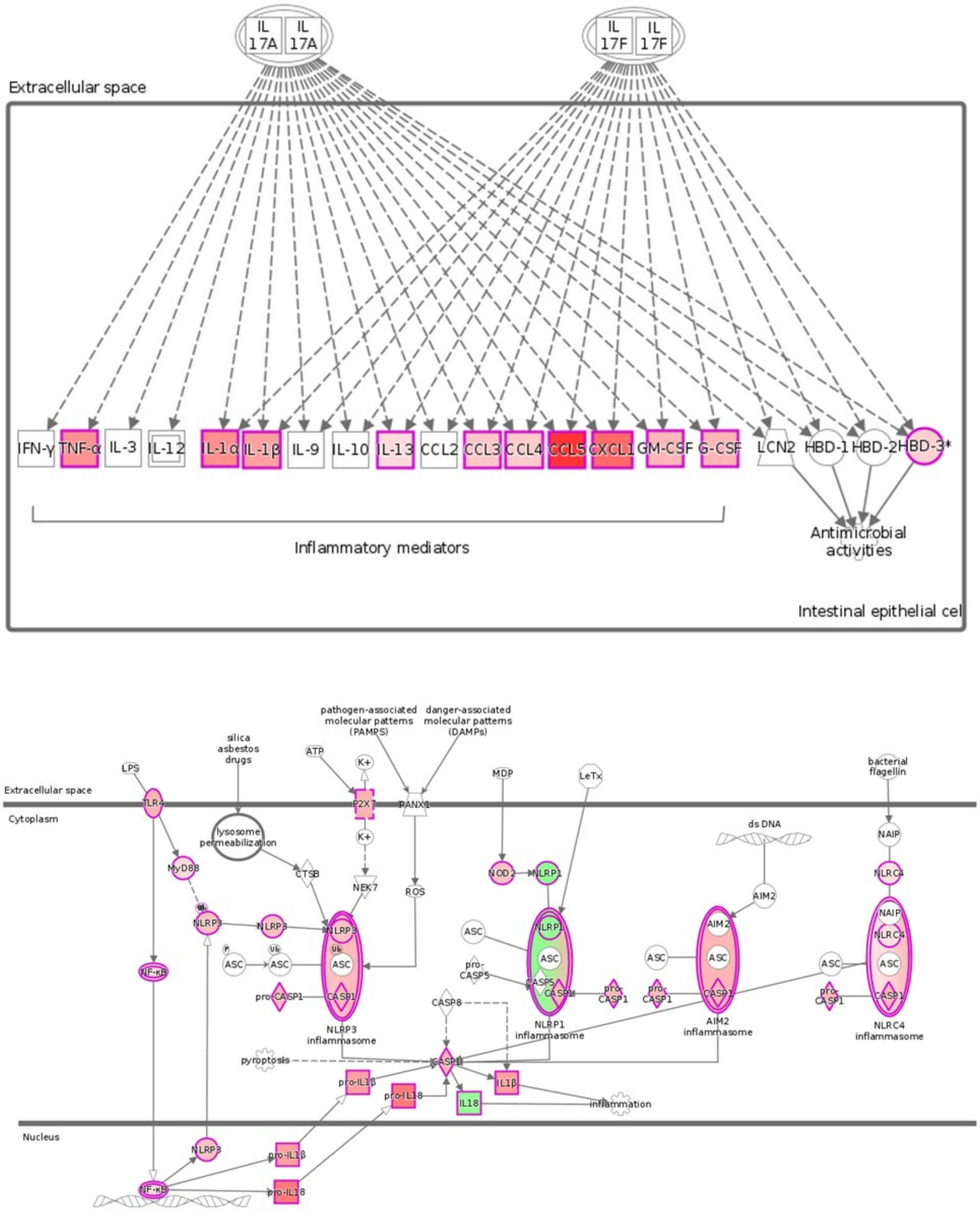

Supplemental Figure S3. Transcriptome and pathway analyses of infected FRPE cells at 3 dpi. Related to Figure 2. IPA canonical pathway analyses revealed differentially regulated cytokines, interleukins, and immune factors in epithelial cells for the IL-17 (A) and inflammasome pathways (B) [upregulated (red); downregulated (green)].

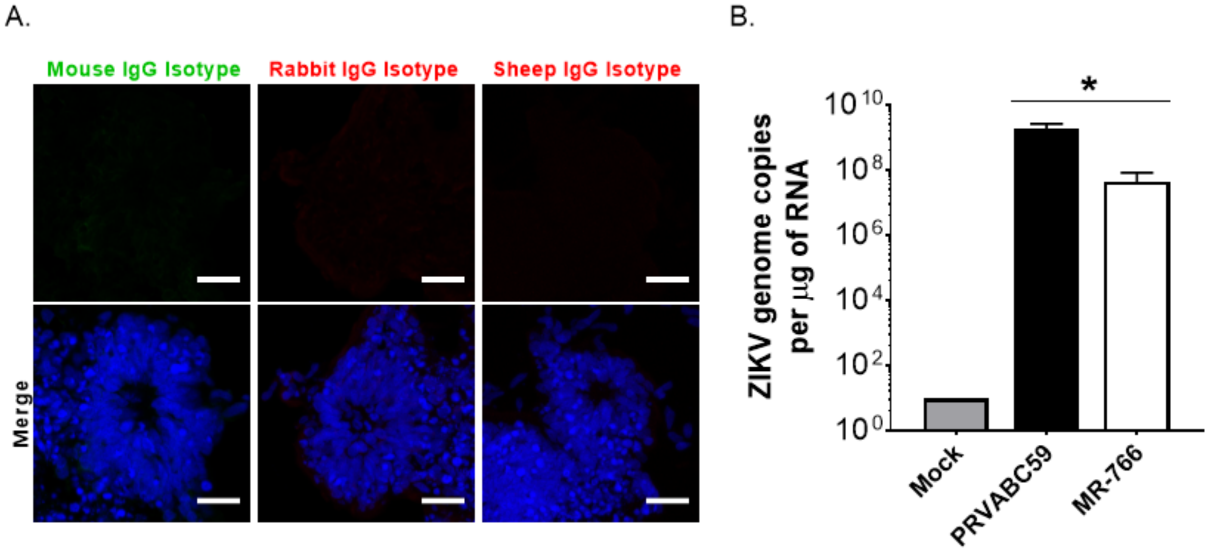

Supplemental Figure S4. Analysis of Retinal organoids and ZIKV infectivity study. Related to Figure 6. (A) Immunofluorescence images of day 34 retinal organoids stained with control IgG isotype antibodies of various species (mouse, rabbit, and sheep) without non-specific background staining. (D) ZIKV genome copies measured in FRPE cells infected with PRVABC59 or MR-766 (MOI 1) at 2 dpi, as measured by RT-qPCR. Students t-test p-value <0.05 (*).

Table S1. List of RT-qPCR primers used in this study. Related to Figures 1, 2, 5, and 6; Figure S1 and S4.

Table S2. List of differentially expressed genes in ZIKV infected FRPE cells at 2 dpi as compared to mock. Ingenuity pathway analyses and disease and function analyses are included. Related to Figure 2.

Table S3. List of differentially expressed genes in ZIKV infected FRPE cells at 3 dpi as compared to mock. Ingenuity pathway analyses and disease and function analyses are included. Related to Figure 2.

Table S4. List of differentially expressed proteins in ZIKV infected FRPE cells at 2 and 4 dpi. Related to Figure 3.

Table S5. List of antibodies used in this study. Related to Figures 1, 4, 5, 6; Figure S1 and S4.
